# Supplementary figures and images for: Quantifying evolutionary importance of protein sites: A Tale of two measures
Source: PLoS Genet. 2021 Apr 7;17(4):e1009476. doi: 10.1371/journal.pgen.1009476 (PMC8026052; doi:10.1371/journal.pgen.1009476)

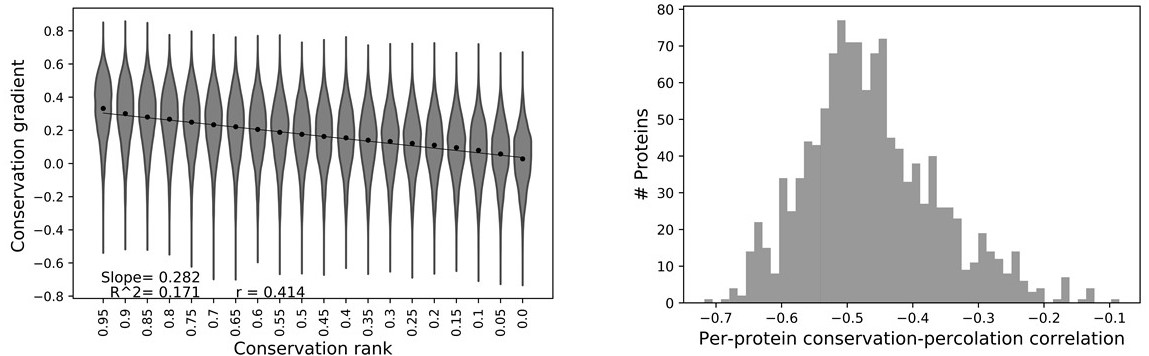

Supplement: S1 Fig — (A) Violin plots and respective average of conservation gradient (calculated as a Spearman correlation between a residue conservation and its distance from a site) as a function of conservation rank for all residues in the dataset binned into 20 equally spaced bins of conservation rank along with the linear fit calculated over all residues. (B) Distribution of per-protein Pearson correlation between residues’ conservation ranks and conservation gradients (conservation gradients calculated as Spearman correlations). (TIF) [file pgen.1009476.s001.tif]

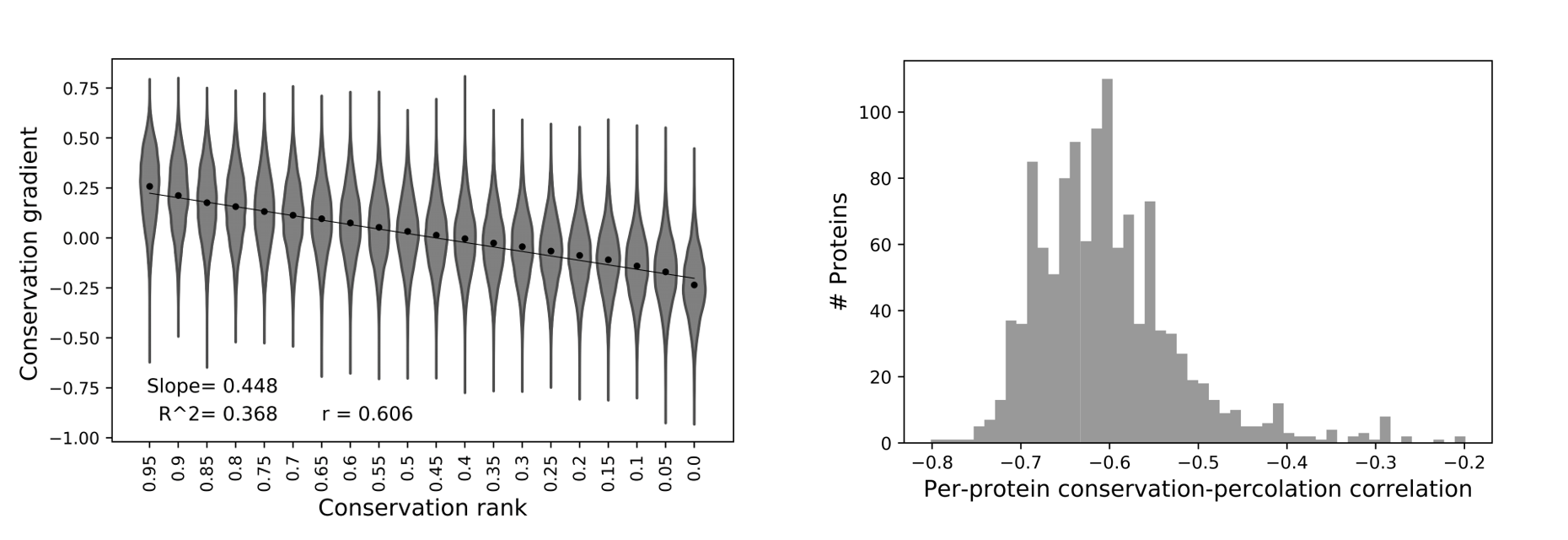

Supplement: S2 Fig — (A) Violin plots and respective average of conservation gradient (calculated as a Pearson correlation between a residue conservation and its distance from a site up to 30Å away) as a function of conservation rank for all residues in the dataset binned into 20 equally spaced bins of conservation rank along with the linear fit calculated over all residues. (B) Distribution of per-protein Pearson correlation between residues’ conservation ranks and conservation gradients (conservation gradients calculated as Pearson correlations up to 30Å away). (TIF) [file pgen.1009476.s002.tif]

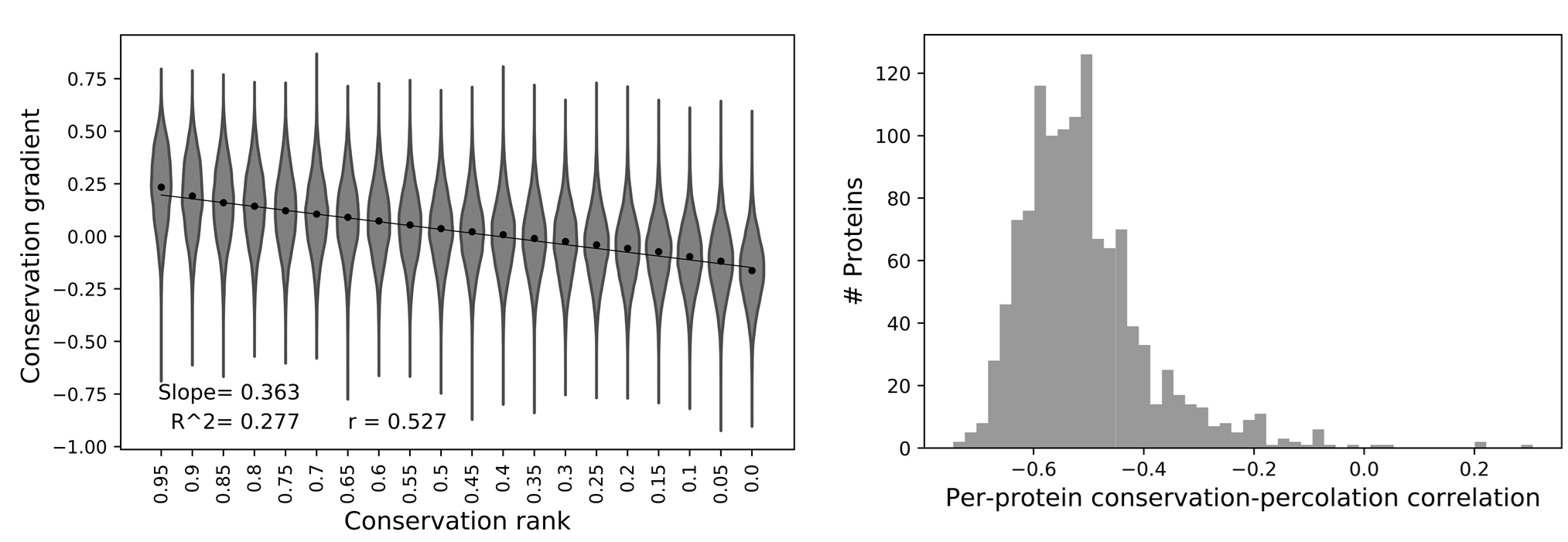

Supplement: S3 Fig — (A) Violin plots and respective average of conservation gradient (calculated as a Pearson correlation between a residue conservation and its distance from a site between 6 Å and 30Å away) as a function of conservation rank for all residues in the dataset binned into 20 equally spaced bins of conservation rank along with the linear fit calculated over all residues. (B) Distribution of per-protein Pearson correlation between residues’ conservation ranks and conservation gradients (conservation gradients calculated as Pearson correlations for between 6 Å and 30Å away). (TIF) [file pgen.1009476.s003.tif]

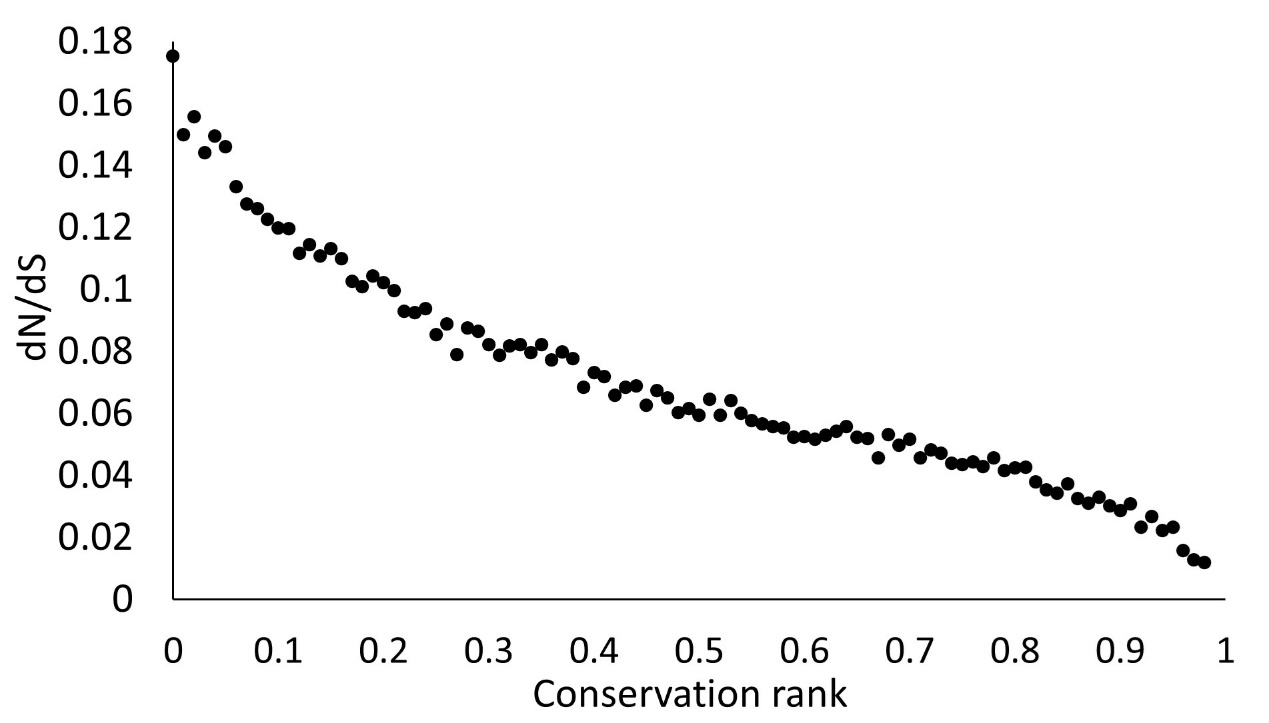

Supplement: S4 Fig — Evolutionary rate (dN/dS) as a function of conservation rank for all residues in the dataset grouped according to their conservation rank and binned into 100 equally spaced bins of conservation rank. (TIF) [file pgen.1009476.s004.tif]

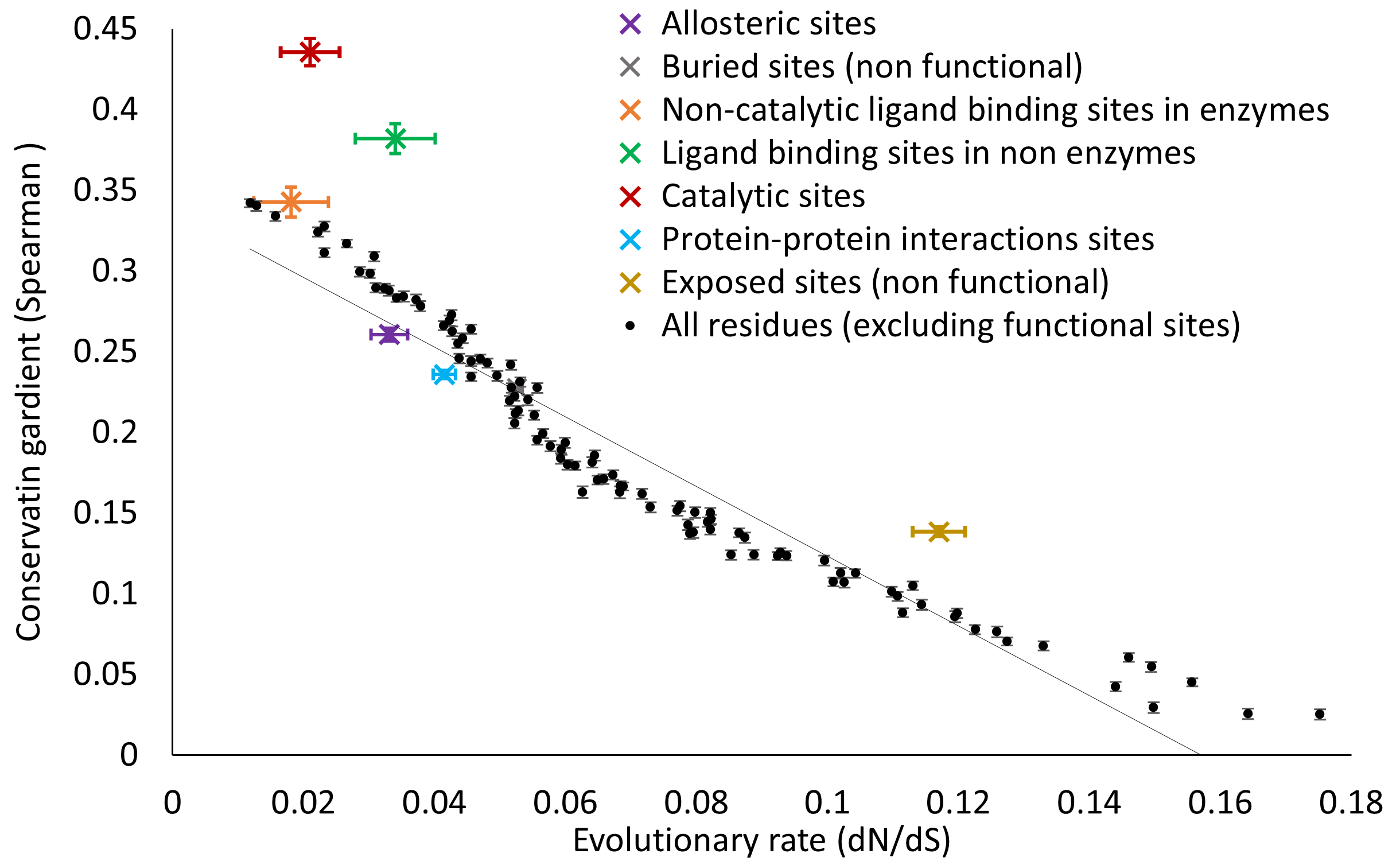

Supplement: S5 Fig — Average conservation gradient (calculated as the average Spearman correlation between conservation of residues and their distance from a site) as a function of the average evolutionary rate (dN/dS) for all yeast protein residues binned according to their annotated conservation rank into 100 equally spaced bins (black) as well as the average conservation gradients of different types of functional sites. (TIF) [file pgen.1009476.s005.tif]

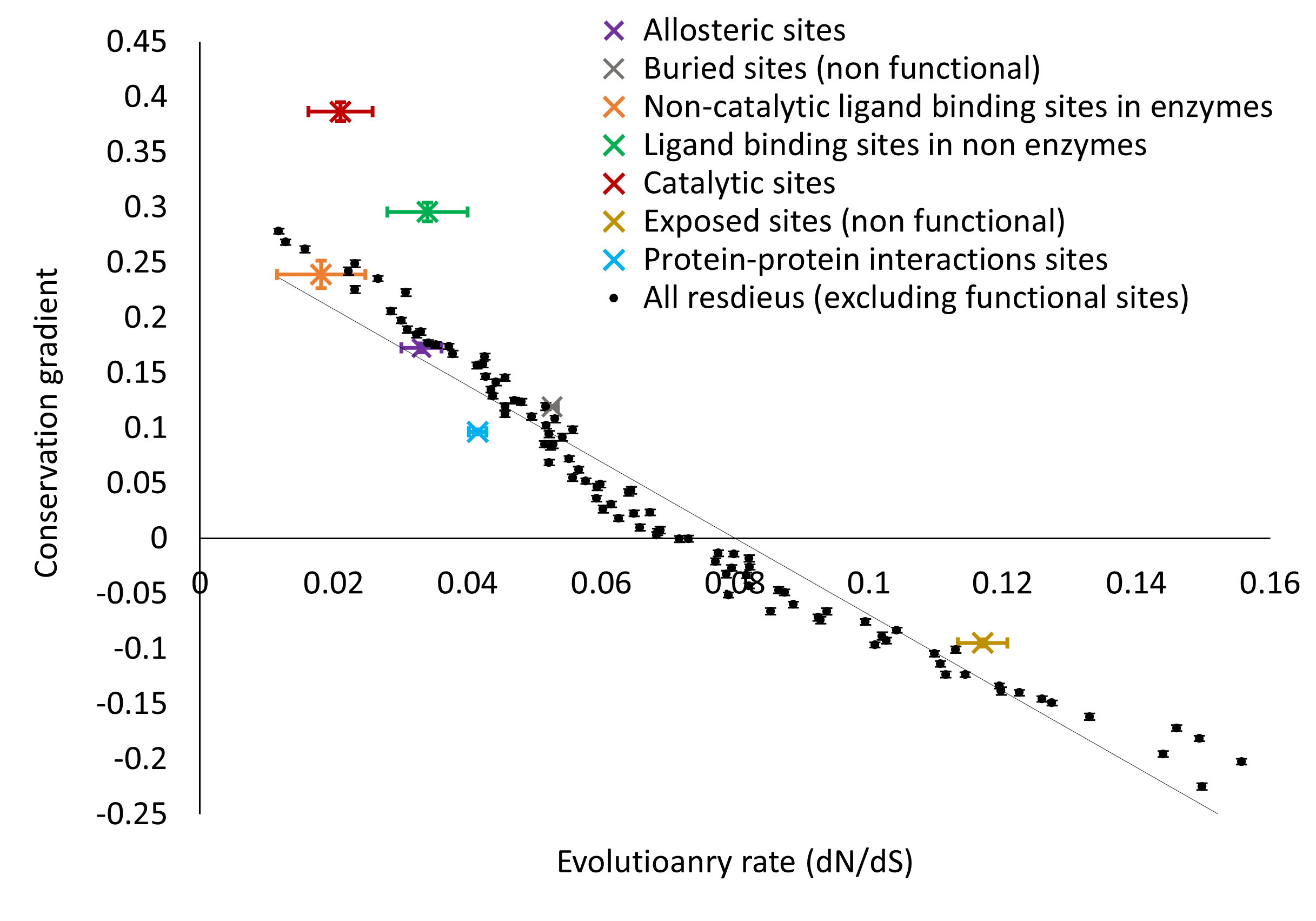

Supplement: S6 Fig — Average conservation gradient (calculated as the average Pearson correlation between conservation of residues and their distance from a site up to 30Å away) as a function of the average evolutionary rate (dN/dS) for all yeast protein residues binned according to their annotated conservation rank into 100 equally spaced bins (black) as well as the average conservation gradients of different types of functional sites. (TIF) [file pgen.1009476.s006.tif]

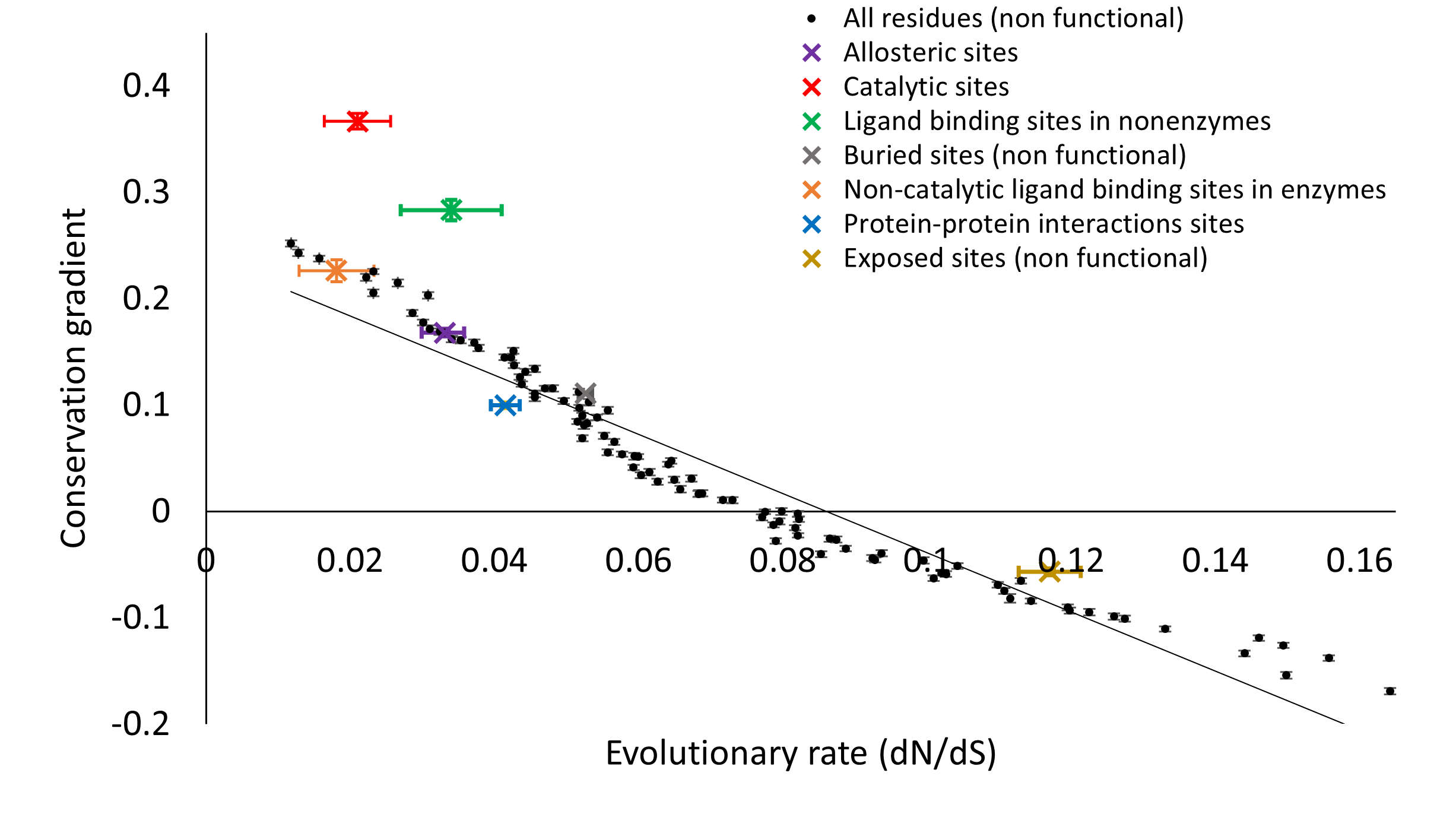

Supplement: S7 Fig — Average conservation gradient (calculated as the average Pearson correlation between conservation of residues and their distance from a site between 6Å and 30Å away) as a function of the average evolutionary rate (dN/dS) for all yeast protein residues binned according to their annotated conservation rank into 100 equally spaced bins (black) as well as the average conservation gradients of different types of functional sites. (TIF) [file pgen.1009476.s007.tif]

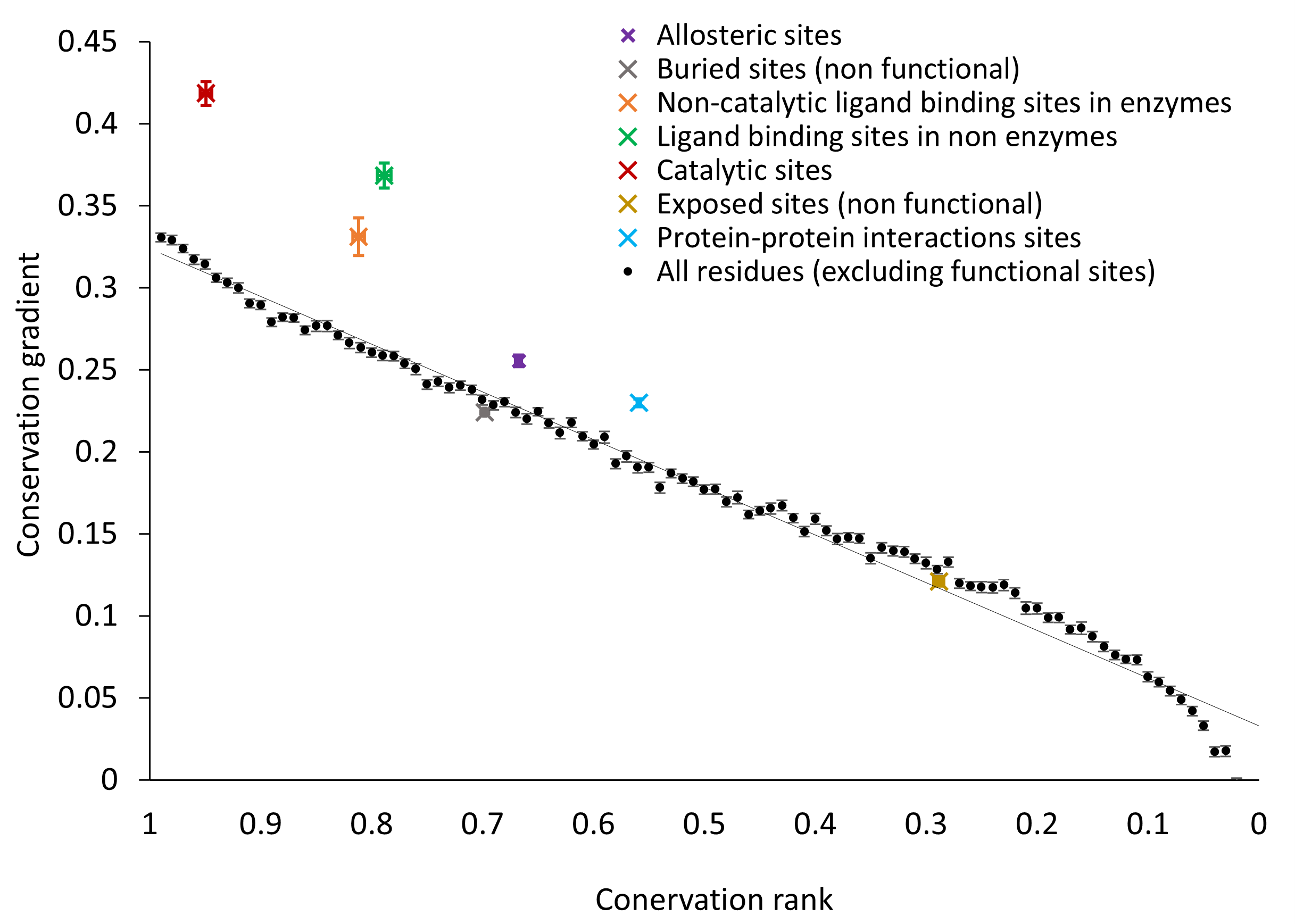

Supplement: S8 Fig — Average conservation gradient as a function of average conservation rank for all yeast protein residues binned into 100 equally spaced bins as well as the average conservation gradients of different types of functional sites. (TIF) [file pgen.1009476.s008.tif]

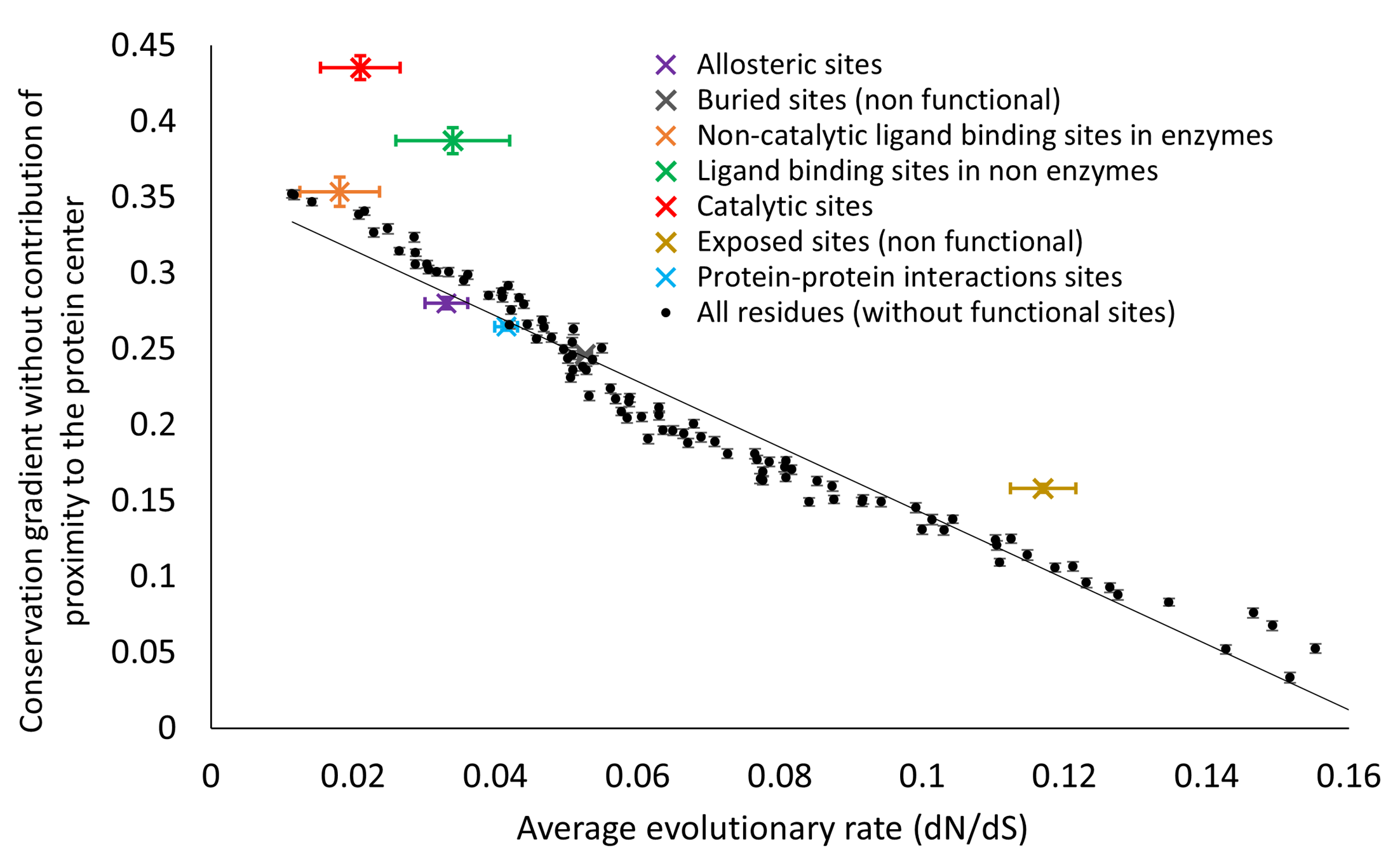

Supplement: S10 Fig — (TIF) [file pgen.1009476.s010.tif]

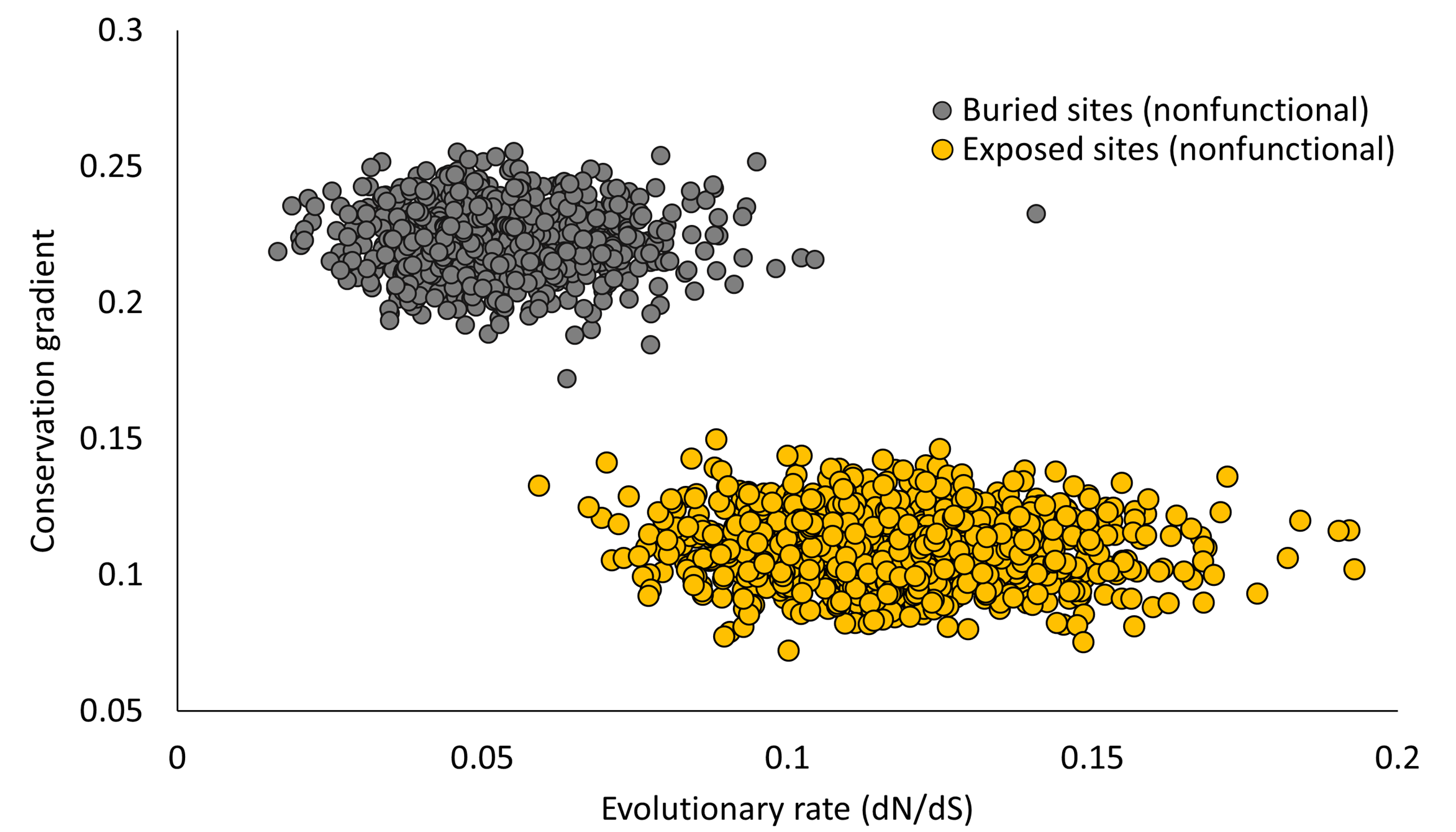

Supplement: S11 Fig — Each circle represents a subset of residues. (TIF) [file pgen.1009476.s011.tif]

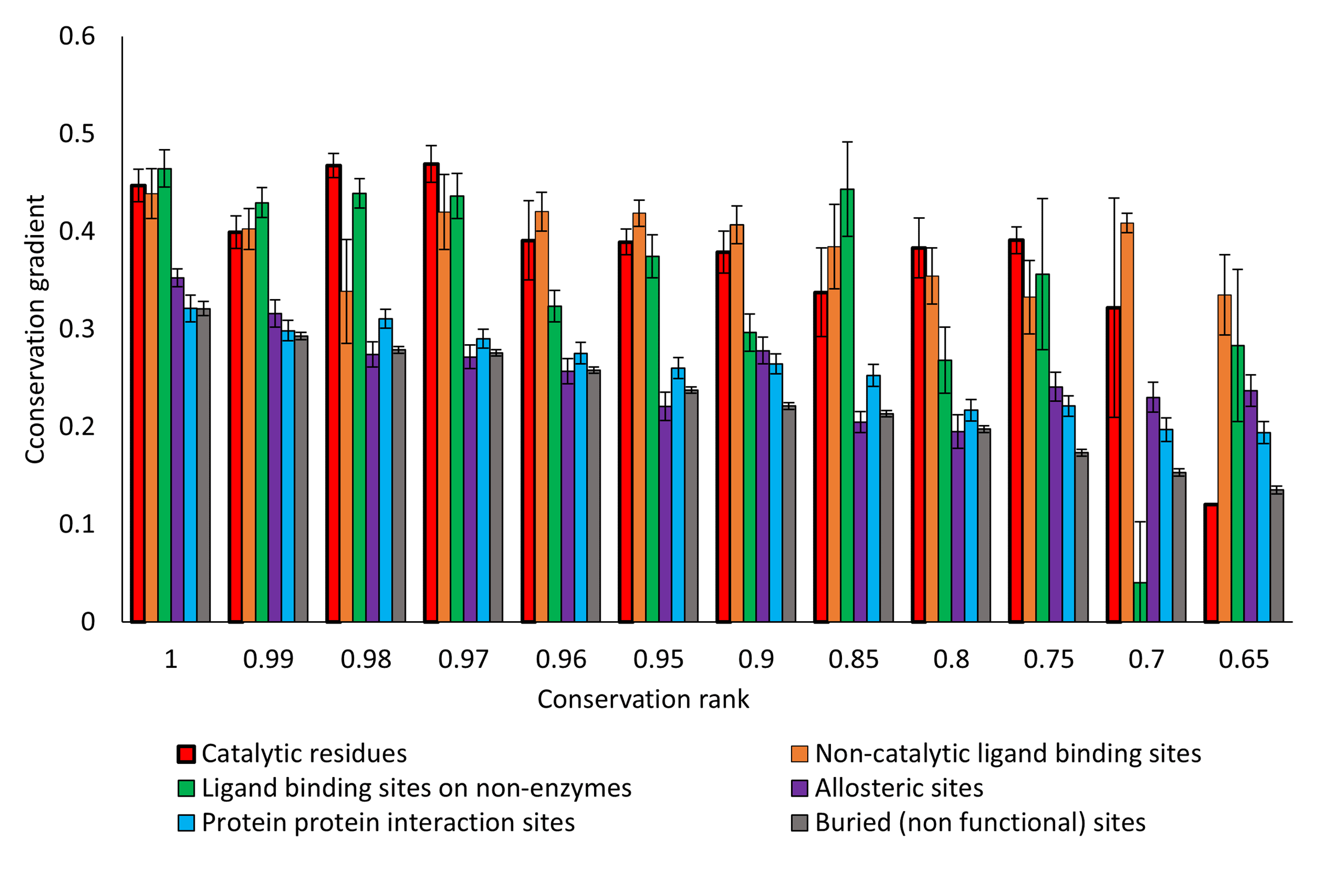

Supplement: S12 Fig — (TIF) [file pgen.1009476.s012.tif]

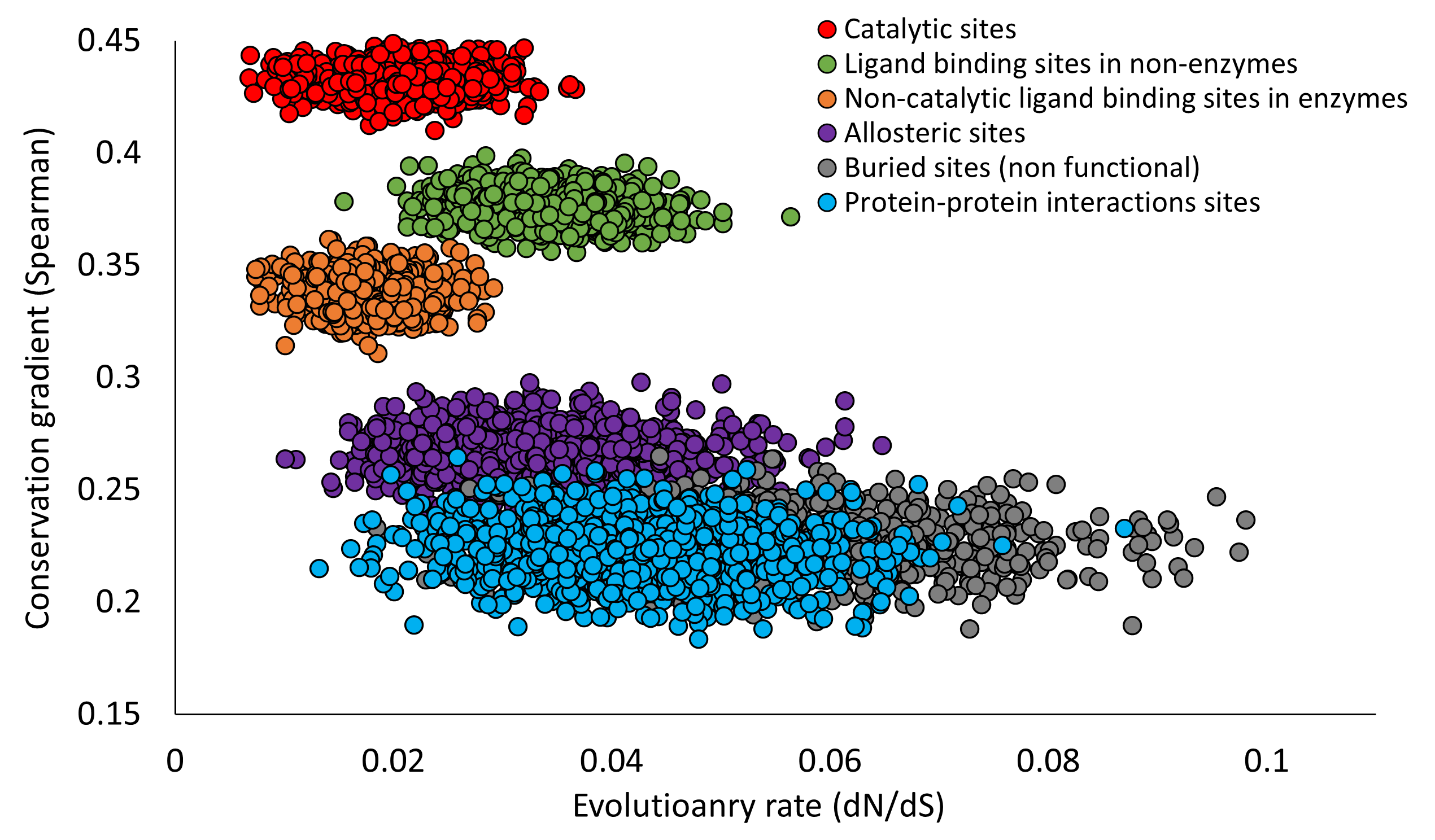

Supplement: S13 Fig — Average conservation gradients (calculated as the average Spearman correlation between conservation of residues and their distance from a site). Each circle represents a subset of residues, coloured by the different types of functional sites. (TIF) [file pgen.1009476.s013.tif]

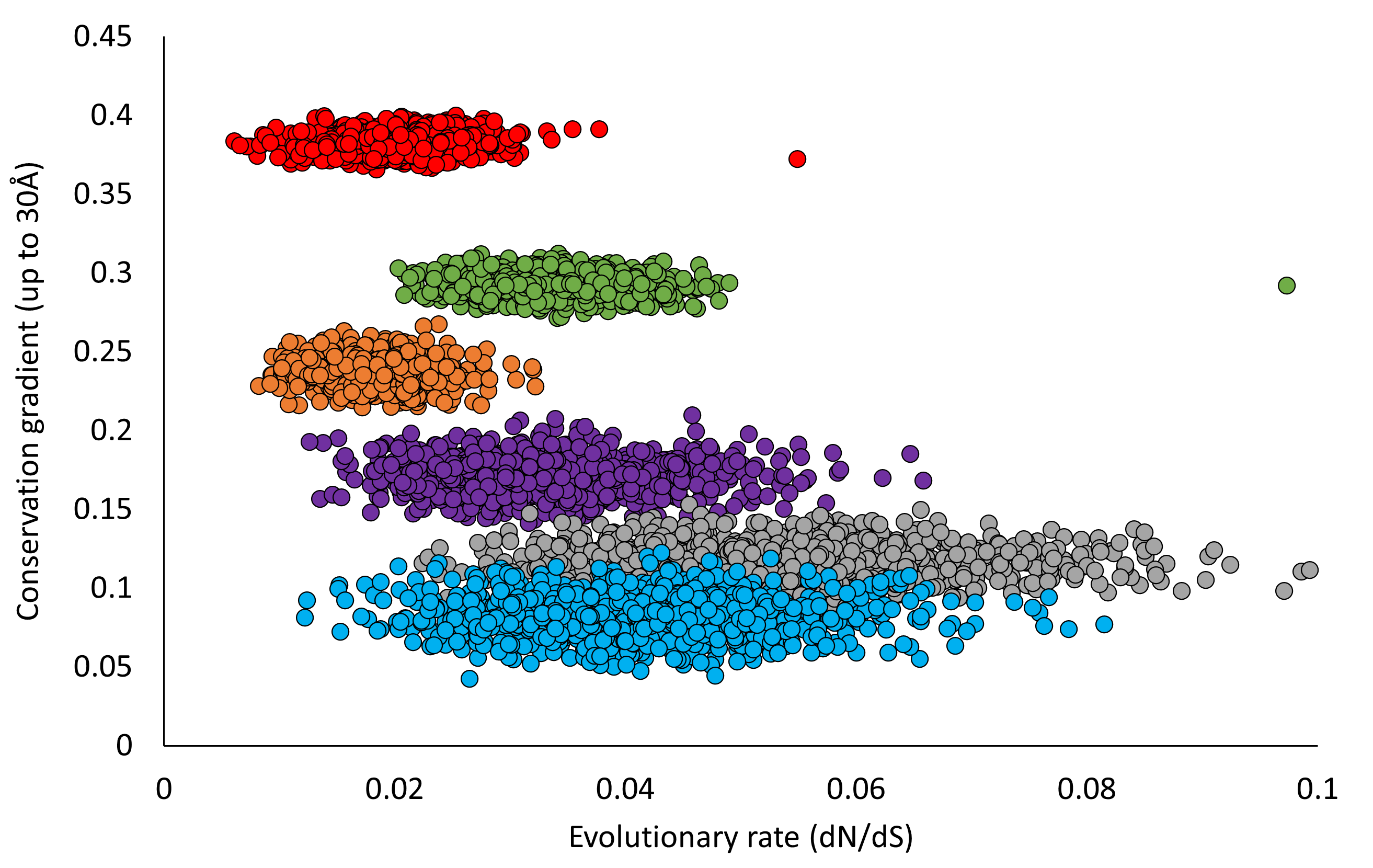

Supplement: S14 Fig — Average conservation gradients (calculated as the average Pearson correlation between conservation of residues and their distance from a site up to 30Å away). Each circle represents a subset of residues, coloured by the different types of functional sites. (TIF) [file pgen.1009476.s014.tif]

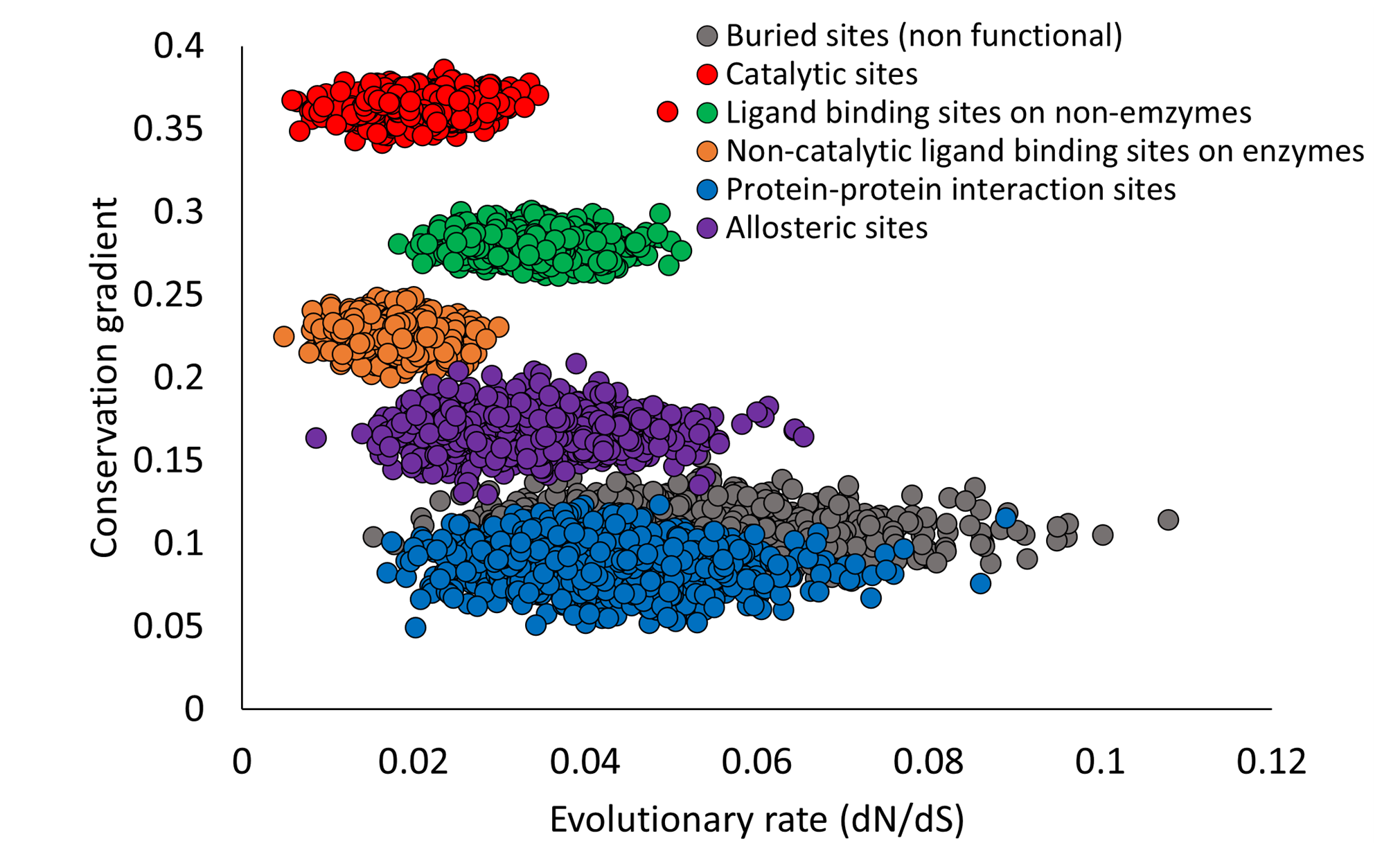

Supplement: S15 Fig — Average conservation gradients (calculated as the average Pearson correlation between conservation of residues and their distance from a site between 6Å and 30Å away). Each circle represents a subset of residues, coloured by the different types of functional sites. (TIF) [file pgen.1009476.s015.tif]

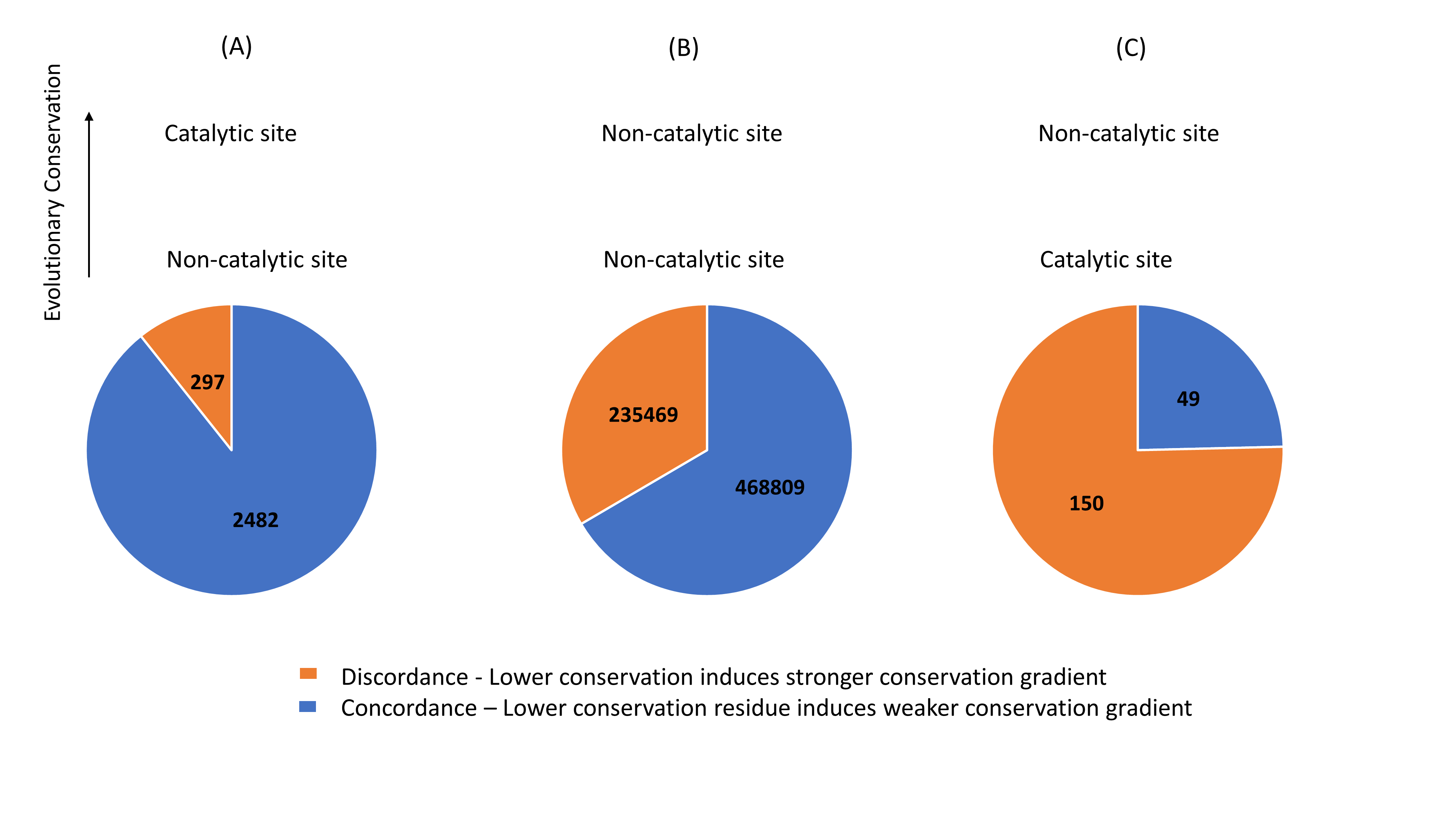

Supplement: S16 Fig — Within the same protein, when conservation gradients are calculated as Spearman correlation between conservation of residues and their distance from a site (A) more conserved catalytic site residues tend to induce stronger conservation gradient than less conserved non-catalytic site residues (binomial test, P<<0.001); (B) more conserved non-catalytic site residues tend to induce stronger conservation gradient than less conserved non-catalytic site residues (binomial test, P <<0.001); (C) less conserved catalytic site residues often induce stronger conservation gradient than more conserved non-catalytic site residues (binomial test, P<<0.001). Functional site residue pairs for which the ordering of residue conservation agrees with the ordering of induced conservation gradient (concordance) are marked in blue. Functional site residue pairs for which the ordering of residue conservation disagrees with the ordering of induced conservation gradient (discordance) are marked in orange. (TIF) [file pgen.1009476.s016.tif]

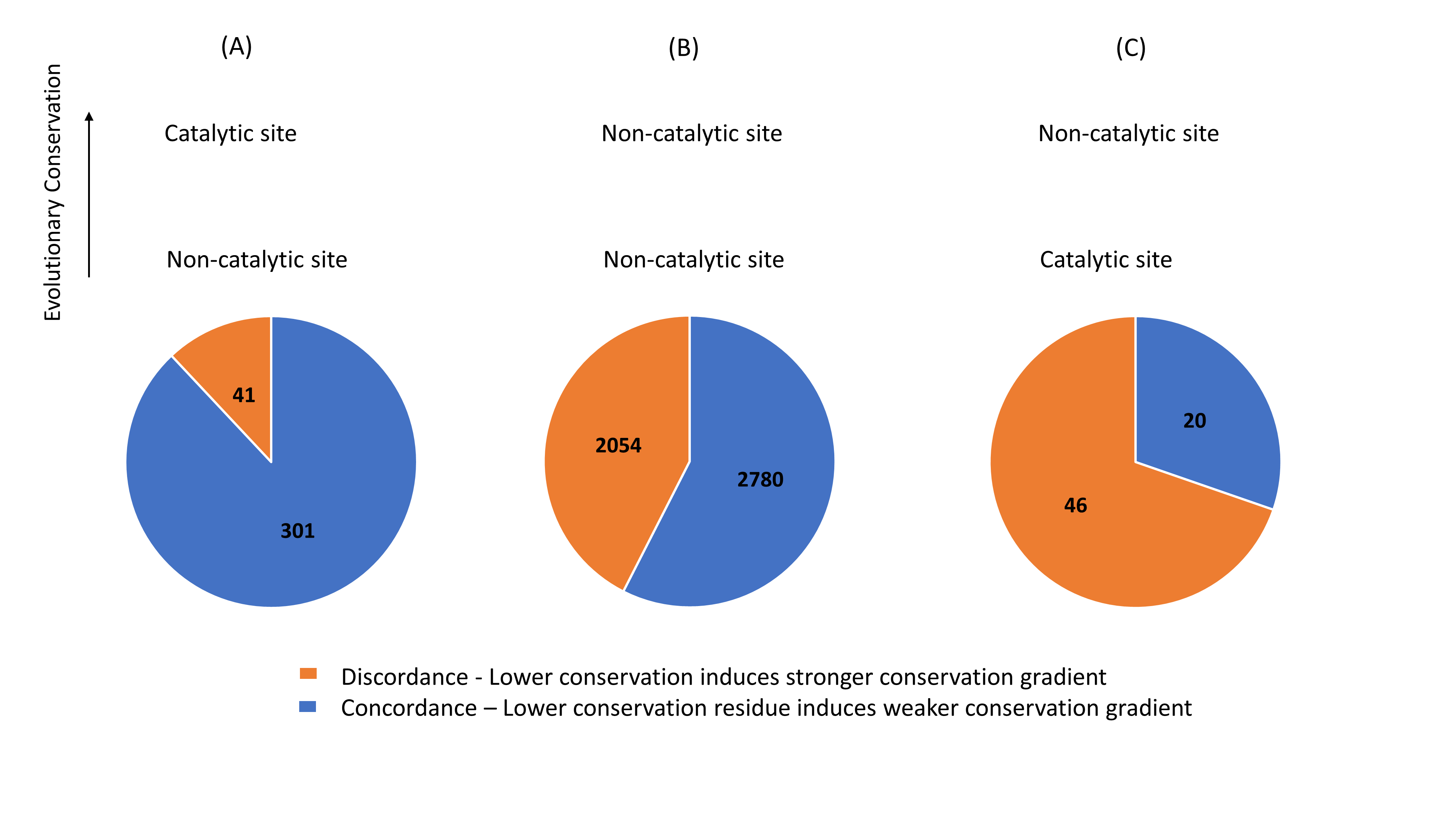

Supplement: S17 Fig — Within the same protein, considering only conservation gradients from the three most-conserved residues within each functional site (A) more conserved catalytic site residues tend to induce stronger conservation gradient than less conserved non-catalytic site residues (binomial test, P<<0.001); (B) more conserved non-catalytic site residues tend to induce stronger conservation gradient than less conserved non-catalytic site residues (binomial test, P <<0.001); (C) less conserved catalytic site residues often induce stronger conservation gradient than more conserved non-catalytic site residues (binomial test, P<0.001). Functional site residue pairs for which the ordering of residue conservation agrees with the ordering of induced conservation gradient (concordance) are marked in blue. Functional site residue pairs for which the ordering of residue conservation disagrees with the ordering of induced conservation gradient (discordance) are marked in orange. (TIF) [file pgen.1009476.s017.tif]

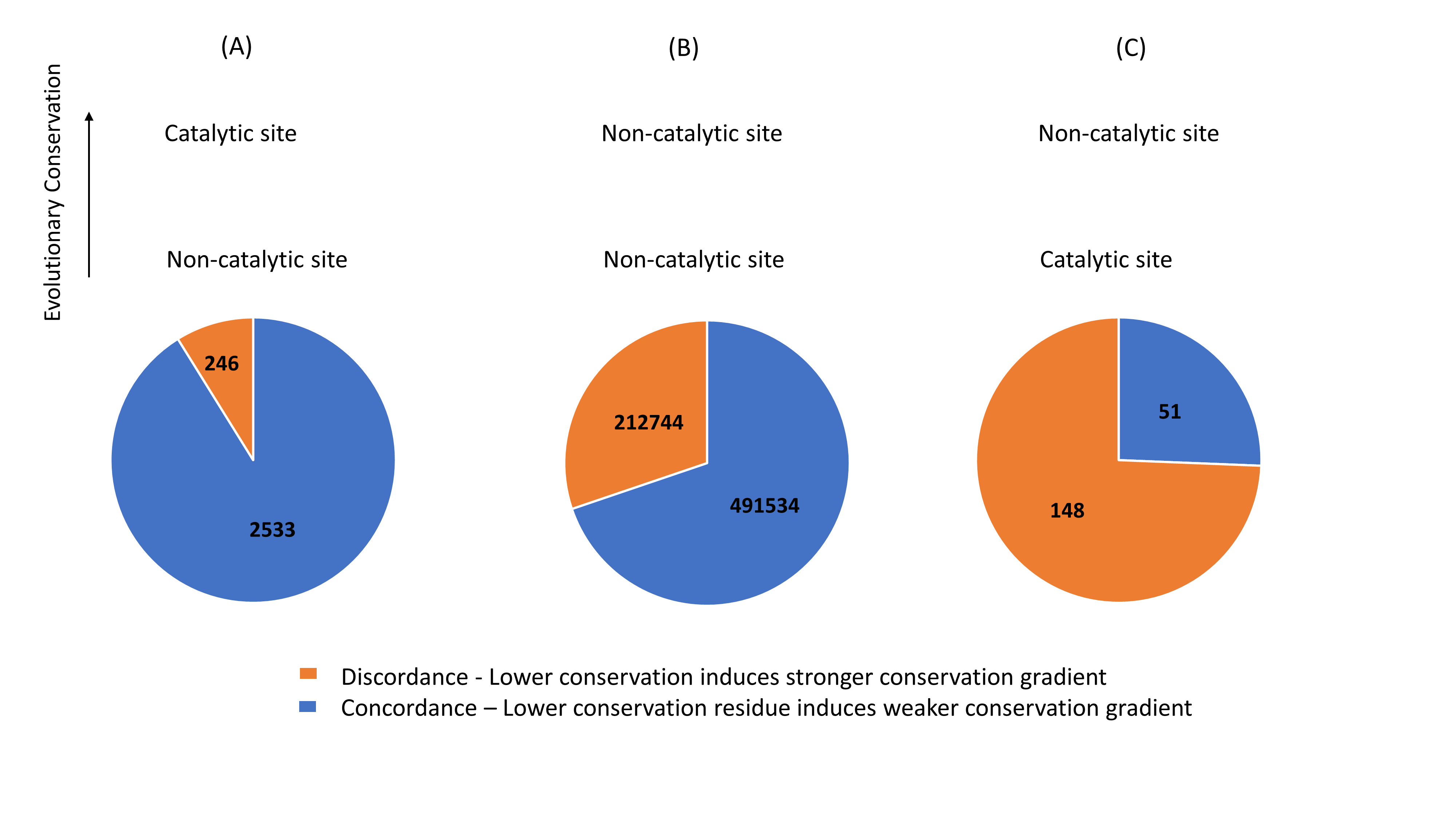

Supplement: S18 Fig — Within the same protein, when conservation gradients are calculated as Pearson correlation between conservation of residues and their distance from a site up to 30Å away (A) more conserved catalytic site residues tend to induce stronger conservation gradient than less conserved non-catalytic site residues (binomial test, P<<0.001); (B) more conserved non-catalytic site residues tend to induce stronger conservation gradient than less conserved non-catalytic site residues (binomial test, P <<0.001); (C) less conserved catalytic site residues often induce stronger conservation gradient than more conserved non-catalytic site residues (binomial test, P<<0.001). Functional site residue pairs for which the ordering of residue conservation agrees with the ordering of induced conservation gradient (concordance) are marked in blue. Functional site residue pairs for which the ordering of residue conservation disagrees with the ordering of induced conservation gradient (discordance) are marked in orange. (TIF) [file pgen.1009476.s018.tif]
