# Supplementary material for: Quantifying evolutionary importance of protein sites: A Tale of two measures
Source: PLoS Genet. 2021 Apr 7;17(4):e1009476. doi: 10.1371/journal.pgen.1009476 (PMC8026052; doi:10.1371/journal.pgen.1009476)
Supplement: Attachment — Submitted filename: Reply to reviewers plos genetics.pdf [file pgen.1009476.s024.pdf]

RESPONSE TO COMMENTS FROM REVIEWER #1

<u>**Reviewer's comments:**</u> **In the manuscript "Quantifying Evolutionary Importance of Protein Sites: A Tale of Two Measures", the authors reported the relationship between the evolutionary constraint of a site in protein and its conservation gradient. For a site, its gradient is the Pearson correlation between the constraints on the other sites and their distances to the focal site. A stronger gradient of a site (larger correlation) indicates that its neighboring sites tend to have stronger constraints than the distal sites. The authors observed a linear relationship, suggesting that the sites with strong constraints also had strong conservation gradients. The authors concluded that such a relationship was likely due to residue-residue contacts among the sites etc. Particularly, the authors found that catalytic sites in enzymes had much stronger conservation gradients than the other sites, and these strong gradients could not be explained by the particularly strong constraints on the focal sites. Therefore, the authors concluded that the observation was likely caused by the catalytic function. Although the results and conclusions are interesting, I have several major concerns.**

<u>**Author's response:**</u> We thank the reviewer for this summary of our work which captures the main points we have tried to convey.

<u>**Reviewer's comments:**</u> **Major points: One main result of the manuscript is that the sites with strong constraints tend to have strong conservation gradients. This linear relationship is convincing and expected. It is known that neighboring residues are expected to be involved in the same biological function e.g. catalysis, binding, and protein stability etc., and thus tend to have similar constraints. For example, in the reference 13, the residues in close proximity to the strongly constrained residues also have strong constraints, whereas the distal residues are less constrained. Therefore, the strongly constrained residues are expected to have relatively larger gradients than the residues with weak constraints, resulting in the observed relationship. However, different from what the author claimed, I think the linear relationship is likely quite weak, given the large variance of each bin. And it worth reporting more details on the regression, e.g. R-square and whether only the median/mean of each bin was used for the regression, which artificially reduces the data variation.**

<u>**Author's response:**</u> We thank the reviewer for this important comment. Violin plots in Fig 1A and Fig 2 describe the distribution of conservation gradients induced from residues as a function of their conservation rank. Following the reviewer suggestion, we added to each plot the correlation coefficient (r), slope and R-square values for the linear correlation between conservation gradients and conservation rank calculated over all the residues without any binning. The correlation coefficient (r) is 0.434 for all residues. Figure legends were changed to better explain that the values of correlation coefficient (r), slope, and R-square are computed over all residues rather than over the mean of each bin. As shown in the plots, the linear correlations between conservation gradients and conservation scores are quite significant and are comparable or even stronger than the conservation gradients themselves.

Reviewer's comments: **Another major result is that the catalytic residues have particularly strong conservation gradients. The authors concluded that the high conservation gradients are probably because the unique requirement for the active site to selectively stabilize the transition state of the catalyzed chemical reaction imposes additional selective constraints on the rest of the enzyme. However, the large gradients of catalytic sites may be because all the identified catalytic sites in a catalytic region have strong constraints, whereas taking the PPI sites as an example, it is known that the PPI interface is relatively large but only a few key PPI residues have strong constraints, and the neighboring sites have weak constraints. This renders even the key residues having low conservation gradients. In addition, catalytic sites tend to be buried rather than exposed on protein surfaces as other binding sites. Located at the core region of a protein further increases conservation gradients due to the paths from core (high constraints) to surface (very low constraints). In sum, without controlling for these factors, the high gradients of the catalytic sites may not be due to the intrinsic catalytic properties.**

Author's response: We thank the reviewer for this comment which helped us refine the manuscript. We have repeated our analysis taking into account only the three most conserved residues from each functional site. We have added these results in Fig 4B as well as Figs S6 and S11 in the Supplementary Material (which correspond to Fig 3A and Fig 5 in the main text). The trend of the results is maintained showing that the most conserved residues within catalytic sites induce significantly stronger conservation gradients compared with the most conserved residues of other functional sites. Notably, this is the case even when the most conserved residues in the non-catalytic sites have similar evolutionary rates to those of the catalytic sites (Fig S6). Therefore, these results support our main conclusion that catalytic sites induce significantly stronger conservation gradients than other sites with similar evolutionary conservation (similar dN/dS). Our conclusion holds when we compare all residues within the entire functional site, as well as when we compare the most conserved residues from each functional site.

Following the reviewer suggestion, we have analyzed the effect of burial/packing of the different functional sites on the conservation gradients induced from them. We utilized the side chain weighted contact number (SC-WCN)(1) as a measure of residue packing. While the average SC-WCN for catalytic sites is higher than the other functional sites, the average SC-WCN for catalytic sites is not significantly different from allosteric sites and ligand binding sites in enzymes (Table 1 in the main text), implying that packing does not dictate the difference in conservation gradients between these sites. Moreover, to examine the differences in conservation gradients between these sites by controlling for the effect of packing, we have constructed a linear regression model for conservation gradients as a function of both their conservation rank and SC-WCN value. We then subtracted the contribution of SC-WCN from the conservation gradient of every residue and plotted the new 'SC-WCN-independent' conservation gradients (Fig 3B). The overall trends and differences in conservation gradients between different types of functional sites are maintained and are not strongly affected by controlling for the contribution of burial/packing.

Moreover, highly conserved buried residues that have similar evolutionary rates as those of catalytic sites, still induce significantly weaker conservation gradients as can be seen in Fig 4A. We therefore conclude that burial/packing of the functional site is not the main cause of the significantly stronger conservation gradients from catalytic sites compared with non-catalytic sites. We have added Table1 and Fig 3B to address these points regarding burial/packing as well as added the above explanations into the main text.

<u>Reviewer's comments:</u> It may be interesting to quantify the influences of all these factors using a simple lattice model. The functional sites, core sites and surface sites have their constraints sampled from respective constraint distributions to calculate gradients. The factors may include the size of the functional region in the protein which has core and surface regions, the average and variance of site constraints in the functional region, the location of the region (surface or core/grove) etc.

<u>Author's response:</u> We agree with the reviewer that quantifying the influence of different factors on conservation gradients using simulations of lattice models could be beneficial. Indeed, several studies have already used lattice models(2) as well as biophysical models(3) to show that catalytic and binding sites induce conservation gradients from them. Hence, further lattice or biophysical modelling studies on the influence of different factors are expected to provide more insight. Our current study is empirical, and we have focused on using available data to support our claims and to study the influence of different factors that the reviewer has suggested on conservation gradients. In future work, it will be interesting to use lattice models with the aim to unify these empirical and theoretical studies.

We have added the following paragraph into the discussion:

"The current study is empirical, using available data on annotated functional sites and their conservation gradient patterns. In future work it will be interesting to use simulation lattice models (2) or biophysical models (3) to examine the effect of different factors on conservation gradient patterns and to unify the empirical and theoretical studies."

<u>Reviewer's comments:</u> Overall, the gradients are moderate or small, calculated using Pearson correlation. Spearman correlation robust to outliers may be necessary to confirm the discovery.

<u>Author's response:</u> We thank the reviewer for this useful comment. We have added Figs S1, S4, S7 & S9 to the Supplementary Material that are plotted using conservation gradients calculated as Spearman correlations and correspond to Figs 1, 3A, 4A & 5 in the main text that use Pearson correlations. Conservation gradients with Spearman correlations are indeed higher compared to Pearson correlations and overall, the trends of the differences in conservation gradients induced from the different functional sites are similar.

__Reviewer's comments:__ **Minor points: The authors mentioned very briefly that their discovery is important to phylogenetic inference, accurate quantification of selective pressure at single-site resolution etc. Please discuss a bit more the details in the discussion.**

__Author's response:__ Following the reviewer's suggestions, we have added more details in the discussion to highlight the relevance of our discovery to accurate quantification of selective pressure at single-site resolution. In addition, we have removed the text regarding phylogenetic inference from the manuscript, as our study is only tangentially related to phylogenetic inference.

__Reviewer's comments:__ **The authors used "long-range" conservation in the manuscript. It would be useful to define the long range e.g. up to 30A. However, there is a possibility that many of the general conservation gradients observed by the authors are mainly due to "short" range residues.**

__Author's response:__ In the initial submission, conservation gradients were calculated over all the residues in the protein domain without distance restriction. Following the reviewer's suggestion, we have repeated our calculations for conservation gradients calculated over residues up to 30Å away from the reference residue. The analysis is presented in Figs S2, S5, S8 & S10 in the Supplementary Material corresponding to Figs 1, 3A, 4A & 5 in the main text. Overall, the trends of the results are maintained. Correlation between conservation rank and induced conservation gradient up to 30Å is actually stronger (Fig S8) than when the gradient is computed over the entire domain. In addition, the difference between conservation gradients induced from catalytic sites and other sites is even more pronounced.

__Reviewer's comments:__ **In this manuscript, many sites from different proteins were pooled together to estimate an average dn/ds for these sites. Many of those sites may have quite different dn/ds. PAML may be used to test whether the sites in a protein have different evolutionary rates, and then estimate the rates respectively for the sites. The multiple groups of sites with different rates may be informative for the analyses.**

__Author's response:__ Indeed, some of the analysis in this manuscript involves grouping together residues from different proteins. In particular, in Fig 3, we group residues with similar conservation rank, as well as residues with the same experimental functional annotation.

In Fig 3, we group residues with similar conservation rank within the protein (using Rate4Site), calculate average dN/dS for each group, and correlate the average dN/dS with conservation gradient. Conservation rank is a per-residue measure which quantifies relative evolutionary rates of different residues within a protein. Indeed, the average dN/dS of the grouped residues is linearly correlated to their average conservation rank (Fig S3), indicating that each group of pooled residues shares similar evolutionary rates. Nonetheless, to account for the possibility that residues within the same conservation rank group may have quite different dN/dS, we additionally calculate the per-residue correlation between conservation

gradient and conservation rank (instead of dN/dS) without any residue grouping (Fig 1A), and our conclusion remains unchanged (i.e., strong correlation between conservation gradient and conservation rank).

In addition, in Fig 3, we group residues with the same experimental functional annotation and calculate their average dN/dS, and correlate the average dN/dS with conservation gradient for each type of functional sites (catalytic sites, allosteric sites, protein-protein interaction sites, etc.). We choose to group residues with the same experimental functional annotation rather than group residues with similar rates of evolution, because our goal is to compare different types of functional sites in terms of their evolutionary behavior. Thus, it makes sense to calculate an average evolutionary rate for each type of functional sites, and then correlate the average evolutionary rate with the average conservation gradient for each type of functional sites (Fig 3).

The reviewer is correct in pointing out that different residues within the same functional annotation group may have very different evolutionary rates, especially if they come from different proteins. To completely neutralize the possible biases arising from grouping residues from different proteins, we additionally carry out a per-protein analysis. We directly compare different functional sites found on the same protein (Fig 5). We show that within the same protein, catalytic sites tend to induce stronger gradients than non-catalytic sites even when they are less conserved. This analysis supports our main conclusion, without having to group residues from different proteins.

As future work it would be interesting to examine further division of sites according to different dN/dS values and analyze conservation gradients from them. This could be useful for the prediction and annotation of new functional sites.

RESPONSE TO COMMENTS FROM REVIEWER #2

<u>Reviewer's comments:</u> **Overall, this is a nice contribution. However, I have one major concern: Most proteins have a natural conservation gradient from the outside to the inside. So any study trying to identify some alternative cause for a conservation gradient must very carefully control for this strong confounder. I don't think the present study does so. I would argue the present study doesn't even properly discuss this issue. To me, the key question is to what extent sites create a conservation gradient given where they are in the protein structure. The authors look at buried and exposed sites, but that's a very crude classification. A site can be buried but relatively close to the surface or right in the center of the protein, and these two sites will experience both different selection pressures and different conservation gradients.**

A good measure to assess how close a site is to the center of the protein core is the weighted contact number (WCN), using an inverse square distance weighting. In fact, WCN is literally a measure of centrality, rather than a measure of number of contacts. (As an aside, many authors in the field mis-understand this issue.) If the authors correlate WCN

with conservation gradient, they should find a fairly strong correlation. Then, the authors can build a regression model that regresses the conservation gradient against both WCN and conservation rank. The degree to which conservation rank contributes to such a model is a measure of the intrinsic conservation rank a site generates, independent of where in the structure it is located. It may well be that if the authors perform this analysis, catalytic sites stand out even more.

<u>**Author's response:**</u> We thank the reviewer for this important comment that helped us refine our manuscript. Following the reviewer's suggestions, we have added the following calculations and corresponding results:

First, we have calculated the average side-chain weighted contact number (SC-WCN) for each type of functional site in our dataset (Table 1). SC-WCN was shown to be the best structural correlate with site-specific evolutionary rates (1). While the average SC-WCN for catalytic sites is higher than the other functional sites, the average SC-WCN for catalytic sites is not significantly different from allosteric sites and ligand binding sites in enzymes (Table 1 in the main text), implying that packing does not dictate the difference in conservation gradients between these sites.

Second, while the overall correlation coefficient between conservation gradients and conservation ranks over all the residues in our dataset is 0.434, the overall correlation coefficient between conservation gradients and SC-WCN values is significantly weaker (0.24). Moreover, to examine the differences in conservation gradients between these sites by controlling for the effect of packing, we have constructed a linear regression model for conservation gradients as a function of both their conservation rank and SC-WCN value. We then subtracted the contribution of SC-WCN from the conservation gradient of every residue and plotted the new 'SC-WCN-independent' conservation gradients (Fig 3B). The overall trends and differences in conservation gradients between different types of functional sites are maintained and are not strongly affected by controlling for the contribution of burial/packing.

Moreover, highly conserved buried residues that have similar evolutionary rates as those of catalytic sites, still induce significantly weaker conservation gradients as can be seen in Fig 4A. We therefore conclude that burial/packing of the functional site is not the main cause of the significantly stronger conservation gradients from catalytic sites compared with non-catalytic sites. We have added Table 1 and Fig 3B to address these points regarding burial/packing as well as added the above explanations into the main text.

<u>**Reviewer's comments:**</u> **Have all data underlying the figures and results presented in the manuscript been provided?**

Large-scale datasets should be made available via a public repository as described in the *PLOS Genetics* <u>data availability policy</u>, and numerical data that underlies graphs or summary statistics should be provided in spreadsheet form as supporting information.

Reviewer #2: No: I think the authors should provide their raw data and analysis scripts. As is, the study is not reproducible.

<u>Author's response:</u> We have added S1 File to the Supplementary Material providing all conservation scores downloaded from ConSurf-DB for all the proteins used in this study. Moreover, calculated conservation gradients for each residue in every protein in the dataset are all found in S2 File. This files also lists the conservation gradients calculated using Spearman correlation, calculated up to 30Å away from the reference residue and calculated when the relative contribution of SC-WCN is eliminated.

In addition, analysis scripts can were deposited to the GiHub repository in the following link: https://github.com/AvitalSharirIvry/Quantifying-Evolutionary-Importance-of-Protein-Sites-A-Tale-of-Two-Measures.git

<u>References</u>

1. Marcos ML, Echave J. Too packed to change: side-chain packing and site-specific substitution rates in protein evolution. PeerJ. 2015 Apr 23;3:e911.
2. Nelson ED, Grishin N V. Evolution of off-lattice model proteins under ligand binding constraints. Phys Rev E. 2016 Aug 15;94(2):022410.
3. Echave J. Beyond Stability Constraints: A Biophysical Model of Enzyme Evolution with Selection on Stability and Activity. Mol Biol Evol. 2019;36(3):613–20.
