# Supplementary material for: Quantifying evolutionary importance of protein sites: A Tale of two measures
Source: PLoS Genet. 2021 Apr 7;17(4):e1009476. doi: 10.1371/journal.pgen.1009476 (PMC8026052; doi:10.1371/journal.pgen.1009476)
Supplement: Attachment — Submitted filename: Response to reviewer PGenetics-D-20-01275R1.pdf [file pgen.1009476.s025.pdf]

RESPONSE TO REVIEWER's COMMENTS

<u>Reviewer's comment:</u> **Reviewer #1: In this revision, the authors added more results and analyses, and the conclusions are more solid. However, there are still several concerns. In the manuscript, the authors emphasize "long-range" gradients. I commented on this before suggesting the authors to define long vs short. In this revision, the authors analyzed the residues within 30A. I feel that to support "long-range", the author should have analyzed the residues beyond a certain cutoff (within a shell) for catalytic residues and other residues.**

<u>Author's response:</u> We thank the reviewer for this comment. As suggested by the reviewer, we have repeated our analysis by calculating the conservation gradient induced by a site using all residues more than 6Å away from the site (but still within 30Å). As shown in Figures S3, S7 and S15, all the trends and conclusions remain the same. The conservation-percolation trend of a linear correlation between conservation of sites and conservation gradients induced from them is valid in these range as well, and catalytic sites are shown to induce stronger conservation gradients than expected in this distance range.

We would like to clarify that the focus of our paper is the comparison between two measures of evolutionary importance for protein sites (namely site-specific conservation versus conservation gradient), rather than the "long-range" nature of the conservation gradient. We therefore agree with the reviewer and have removed the phrase "long-range" from the manuscript.

<u>Reviewer's comment:</u> **I suggested that the strong correlation between constraint and conservation gradient may be due to the location of catalytic residues, i.e. close to the core of proteins. (The core-surface and catalytic function together lead to the high gradient). To address this comment, the authors used the numbers of contact residues to indicate the packing. However, catalytic residues (other ligand binding residues) may be in a groove (thus close to core) but have no contact residues. Number of contact residues may not be a direct measure for this purpose.**

<u>Author's response</u>: We would like to clarify that, in the revised manuscript, we used SC-WCN to measure the degree of packing of a residue within the protein. Here, every other residue in the protein (not just the contact residues) contributes to the SC-WCN value of a given residue, with the contribution inversely proportional to the square of the distance. Hence, residues close to the center of the protein (including residues in a groove) will have higher SC-WCN values than other residues.

Following the reviewer's suggestion, in addition to testing the correlation between the SC-WCN value of a protein site and its conservation gradient, we have also tested the correlation between the location of a protein site ("core-proximity", i.e., its distance to the center of the protein) and its conservation gradient. The center of the protein is defined as the residue which minimizes the harmonic mean of its squared distance to all other residues in the protein.

Indeed, as the reviewer suggested, the average distance of catalytic sites to the protein center is shorter than that of other functional sites (Table 1in the main text), as expected. In addition, Pearson correlation between distance to the protein center and conservation gradient of a site is -0.18, which is significant (p-value<<0.05). While this indicates that the location of the functional site in the protein structure contributes significantly to its conservation gradient, this correlation is still significantly smaller than the correlation between conservation rank and conservation gradient which is 0.43, implying that the distance to the protein center does not completely explain the differences in conservation gradients between functional sites. In addition, non-functional buried sites (which are closer to the protein center) induce significantly lower conservation gradients on average compared to allosteric sites and protein-protein interaction sites (which are further away from the protein center).

To examine the differences in conservation gradients between functional sites by controlling for the effect of their distance to the protein center, we have constructed a linear regression model for conservation gradients as a function of both their conservation rank as well as their distance to the protein center. We then subtracted the contribution of distance to protein center ("core-proximity") from the conservation gradient of every residue and plotted the new "core-proximity-independent" conservation gradients (S10 Fig). Our findings and conclusions remain the same. Specifically, catalytic sites are still shown to induce significantly stronger conservation gradients than expected by the conservation-percolation trend, even when the contribution of distance to protein center ("core-proximity") is eliminated.

We therefore conclude that the distance of the functional site to the protein center is not the main cause of the significantly stronger conservation gradients from catalytic sites compared with non-catalytic sites. We have added the data into Table1 and S10 Fig to address these points as well as added the above explanations into the main text.

Reviewer's comment: **Fig2 shows the relationship between the normalized conservation ranks of residues and their conservation gradients, for different types of residues, such as ligand binding sites, catalytic sites, allosteric sites etc. The conclusion is that the relationship for catalytic sites is quite unique. However, from the fig, it seems that this is likely because the catalytic sites have x range 0.95 to 0.65. For some other residue types, the relationships in this range seem similar to that of catalytic sites. The authors may need to add regression lines using only that x range. The r-square (cor squared) is quite small, indicating the fitting is at most moderate.**

Author's response: We thank the reviewer for this comment, and we would like to take this opportunity to clarify the two main points of the paper. The first point of the paper (conveyed in Figures 1 & 2) is that there is a universal linear relationship between site-specific conservation and site-induced conservation gradient. The second point (conveyed in Figures 3, 4 & 5) is that catalytic residues, while still obeying this universal linear trend, induce conservation gradients which are stronger than expected by this linear trend.

Figures 1 & 2 convey the first point of the paper, showing that the linear relationship between site-specific conservation and site-induced conservation gradient is universal

and applies to all types of sites, including catalytic sites. It is true that the percentage of highly conserved residues (with conservation rank > 0.65) in catalytic sites is much higher than in other functional sites, making it more difficult to see the linear trend for catalytic sites. Although small, the correlation between site-specific conservation rank and site-induced conservation gradient for catalytic residues is statistically significant.

We agree with the reviewer that it is more accurate to compare all functional sites on the same x-range of above 0.6. We added the R2, R and slope values in a S1 Table in the supplementary material. Our findings and conclusions remain unchanged, namely that the linear relationship between site-specific conservation and site-induced conservation gradient is universal and applies to all types of sites.

Reviewer comment: **For fig4, are the black dots "all residues (w/o functional sites)"? Their results are missing in panel B. The result of such residues can tell how much the constraints on residues alone influence the gradients.**

Author's response: The grey-black dots in Figure 4A are the buried non-functional residues which were indeed missing in Figure 4B by accident. In this revision, we have added the grey-black dots for buried non-functional residues back in Figure 4B. Furthermore, we have done the same calculations for exposed non-functional residues and presented them in Figure S11 in the supplementary material. These figures (Figures 4 and S11) support our main conclusion that catalytic sites induce stronger conservation gradient on average than other functional and non-functional sites with similar levels of site-specific conservation.

Reviewer's comment: **Conservation gradients depend on the relative residue constraints within each protein. The normalization used by fig1&2 is more reasonable than comparing dn/ds from different proteins. It seems that the normalized conservations can be used for those key analyses in fig3&4 with x changed accordingly.**

Author's response: We have repeated the analysis in Figures 3 & 4 with the x-axis changed to the ConSurf-based conservation rank (Figures S8 and S12 in the Supplementary Material). These new figures (Figures S8 and S12) are in broad agreement with Figures 3 and 4, and together they support our main conclusion that catalytic sites induce stronger conservation gradient on average than other functional and non-functional sites, even after controlling for site-specific conservation level. In addition to catalytic sites, other ligand binding sites also exhibit somewhat higher conservation gradient than allosteric sites and protein-protein interaction sites, likely due to hidden, unannotated catalytic sites in our dataset of ligand binding sites.

Reviewer's comment: **About the conclusion in discussion, I think the measures in this manuscript probably can not be informative for "de-novo functional site prediction and protein design", because many functional sites, except catalytic sites, are similar to non-functional sites in terms of the measures.**

Authors response: This sentence is now deleted from the discussion.

Reviewer comment: **The following sentences may contain typos.**
When considering only the three most conserved residues from each functional site (Fig 4B), subset of residues exhibits lower evolutionary rates and higher conservation gradients compared with subsets from all functional sites residues (Fig 4A).
Beyond the classical, "intrinsic" measure of conservation and the "extrinsic" measure which is conservation gradient the site exerts on the rest of the protein.

Author's response: We have fixed the typos in the manuscript.
